# Knowledge, health beliefs and attitudes towards dementia and dementia risk reduction among the Dutch general population: a cross-sectional study

**DOI:** 10.1186/s12889-021-10913-7

**Published:** 2021-05-03

**Authors:** J. Vrijsen, T. F. Matulessij, T. Joxhorst, S. E. de Rooij, N. Smidt

**Affiliations:** 1 Department of Epidemiology, University of Groningen, University Medical Centre Groningen, Hanzeplein 1, PO Box 30 001, FA40, 9700 RB Groningen, the Netherlands; 2 Department of Internal Medicine, University of Groningen, University Medical Centre Groningen, Groningen, the Netherlands

**Keywords:** Dementia, Knowledge, Health beliefs, Risk reduction behaviour, Survey

## Abstract

**Background:**

Positive health beliefs and attitudes towards dementia and dementia risk reduction may encourage adopting a healthy behaviour. Therefore, we aimed to investigate the knowledge, health beliefs and attitudes towards dementia and dementia risk reduction among the Dutch general population and its association with the intention to change health behaviours.

**Methods:**

A random sample of Dutch residents (30 to 80 years) was invited to complete an online survey. We collected data on knowledge, health beliefs and attitudes towards dementia (risk reduction) and the intention to change health behaviours. Multivariable logistic regression analyses were used to obtain effect estimates.

**Results:**

Six hundred fifty-five participants completed the survey. In general, participants had insufficient knowledge about dementia and dementia risk reduction. Participants had relatively high scores on general health motivation and perceived benefits, but low scores on perceived susceptibility, perceived severity, perceived barriers, cues to action and self-efficacy. Individuals with higher scores on perceived benefits and cues to action had more often the intention to change their behaviour with regard to physical activity (OR = 1.33, 95%-CI:1.11–1.58; OR = 1.13, 95%-CI:1.03–1.24, respectively) and alcohol consumption (OR = 1.30, 95%-CI:1.00–1.69; OR = 1.17, 95%-CI:1.02–1.35, respectively). Younger excessive alcohol consumers with higher perceived severity scores had more often the intention to change their alcohol consumption behaviour (OR = 2.70, 95%-CI:1.04–6.97) compared to older excessive alcohol consumers. Opposite results were found for middle-aged excessive alcohol consumers (OR = 0.81, 95%-CI:0.67–0.99). Individuals who perceived more barriers had more often the intention to change their diet (OR = 1.10, 95%-CI:1.01–1.21), but less often the intention to change their smoking behaviour (OR = 0.78, 95%-CI:0.63–0.98). Moreover, less educated individuals with higher perceived benefits scores had less often the intention to change their diet (OR = 0.78, 95%-CI:0.60–0.99), while highly educated individuals with higher perceived benefits scores had more often the intention to change their diet (OR = 1.41, 95%-CI:1.12–1.78).

**Conclusions:**

The knowledge, beliefs and attitudes towards dementia and dementia risk reduction among the Dutch general population is insufficient to support dementia risk reduction. More education about dementia and dementia risk reduction is needed to improve health beliefs and attitudes towards dementia and dementia risk reduction in order to change health behaviour.

**Supplementary Information:**

The online version contains supplementary material available at 10.1186/s12889-021-10913-7.

## Introduction

Dementia is one of the fastest growing health problems in the world. Currently, around 50 million people are living with dementia worldwide and due to the aging population this number will increase to an imposing 152 million in 2050 [1]. Also in the Netherlands, it is expected that the number of people suffering from dementia will increase from 280,000 people in 2018 to more than 620,000 in 2050 [2]. However, delaying the onset or progression of dementia could help to tackle these increasing prevalence rates. Therefore, the World Health Organization set up a global action plan which includes multiple actions such as making dementia a public health priority worldwide, increase dementia awareness and reduce the risk of dementia [3].

Livingston et al.(2020) found that 40% of all dementia cases worldwide are attributable to 12 modifiable risk factors, including less education, hearing loss, midlife hypertension, midlife obesity, smoking, depression, physical inactivity, diabetes, low social contact, excessive alcohol consumption, traumatic brain injury, and air pollution [4, 5]. However, despite the large potential for prevention, previous research showed that most people have little knowledge of these modifiable risk factors and the possibility to reduce their risk of dementia [6–8]. Furthermore, changing health behaviour is difficult and complex [9].

A number of health behaviour models were developed in order to understand health behaviour and the determinants of health behaviour change (e.g., health belief model (HBM), Trans Theoretical Model) [10, 11]. Subsequently, these models contributed to the development of the Integrated Change model, which assumes that the process of health behaviour change can be distinguished in three phases: 1) Awareness, 2) Motivation and 3) Action [12, 13]. In the first phase, individuals need to become aware of their unhealthy behaviours, where important factors are derived from the HBM, such as an individual’s subjective risk assessment of getting a condition, how serious this condition and its consequences are and cues to action [10]. In the motivation phase, individuals need to become motivated to change health behaviour, where factors as the perceived benefits of health behaviour change, social influence and the confidence in being able to perform the desired behaviour are important. Subsequently, an intention to change health behaviour is formed [11]. In the last phase, depending on the perceived barriers, this intention to change health behaviour is leading to actual health behaviour change by conducting preparatory actions [12, 13].

Two studies examined the health beliefs and attitudes towards dementia (risk reduction) using the Motivation to Change Lifestyle and Health Behaviours for Dementia Risk Reduction (MCLHB-DRR) scale among the Australian (50 years and older) and Turkish (40 years and older) population [14, 15]. Akyol et al. (2020) found that males had lower perceived severity and cues to action scores and higher perceived barriers scores compared to females. Older individuals had lower perceived benefits, cues to action and self-efficacy scores compared to younger individuals. Furthermore, less educated individuals had lower perceived benefits and self-efficacy scores and higher perceived barriers scores [15]. However, Kim et al. (2014) only found significant age differences in males, but not in females [14]. Furthermore, a few studies conducted in Australia and the United States of America investigated how these health beliefs influence the intention to engage in dementia risk reduction behaviours and showed that age, perceived benefits and barriers, self-efficacy and knowledge about dementia risk reduction are associated with the intention to adopt a healthy lifestyle for dementia risk reduction in general [16, 17].

To our knowledge, no research is conducted to examine the knowledge, health beliefs and attitudes towards dementia (risk reduction) in the Netherlands and its association with the intention to change individual health behaviours. Therefore, the aim of this study was firstly, to investigate the knowledge, health beliefs and attitudes towards dementia (risk reduction) among the Dutch general population, secondly to what extent the knowledge, health beliefs and attitudes differ between demographic subgroups and finally, to investigate the association between these determinants and the intention to change health behaviours.

## Method

### Study design and participants recruitment

This cross-sectional study was conducted from July to September 2018. The study population consisted of residents of the municipality of Groningen aged between 30 and 80 years. A random sample of 4500 residents stratified for age (30–39, 40–49, 50–59, 60–69 and 70–80 years old) and gender (for each strata, 450 males and 450 females) were randomly selected by the municipality of Groningen, taken a response rate of 12% into account. This enables us to obtain a sample that represents the entire population being studied, making sure that each subgroup is represented in the study. The selected 4500 residents were invited by a letter, which included a web address giving access to the online survey ‘Lifestyle and dementia’. As the questionnaire was in Dutch, participants were required to be able to read the Dutch language. This study was assessed and approved by the Medical Ethical Committee of the University Medical Centre Groningen (METc2018/123). All participants provided informed consent. All methods were performed in accordance with the Declaration of Helsinki.

### Data collection

#### Knowledge about dementia risk reduction

For the assessment of the knowledge regarding dementia (risk reduction), the Dementia Knowledge Assessment Scale (DKAS) was included in the survey [18]***.*** The original DKAS scale consists of 25 items covering four subscales: Causes and Characteristics (7 items), Communication and Behaviour (6 items), Care Considerations (6 items) and Risk Factors and Health Promotion (6 items) [18]. We translated the original DKAS scale into the Dutch language using the method of Beaton et al. (2000) [19], and subsequently investigated the validity of the Dutch version of the DKAS. After cross-cultural validation, the Dutch version of the DKAS scale showed not to be valid to measure the knowledge about dementia in the Dutch general population (see Additional file 1: Appendix 1). Therefore, we decided to only use the individual items of the DKAS with good face and content validity that are quite similar to the items used in previous studies [20, 21].

#### Health beliefs and attitudes towards dementia risk reduction

For the assessment of health beliefs and attitudes towards dementia (risk reduction), the Dutch version of the MCLHB-DRR scale was used [22]. The original MCLHB-DRR scale was developed by Kim et al.(2014) [14], which was translated into Dutch and cross-culturally validated by Joxhorst et al.(2020) [22]. The Dutch version of the MCLHB-DRR consists of 23 items reflecting 7 subscales, namely perceived susceptibility (3 items), perceived severity (5 items), perceived benefits (2 items), perceived barriers (4 items), cues to action (4 items), general health motivation (3 items) and self-efficacy (2 items). The items are rated on a 5-point Likert scale from strongly disagree (1 point) to strongly agree (5 points). Higher subscale scores indicate more positive beliefs and attitudes towards changing health behaviour for dementia risk reduction. To enable a straightforward interpretation and comparison between subscales with different number of items, subscale scores were also transformed to a 100-point scale.

#### Intention to change health behaviours

To assess the intention to change health behavioural-related risk factors for dementia (e.g., physical inactivity, poor-to-moderate adherence to a Mediterranean diet, excessive alcohol consumption and smoking), participants were asked to indicate their ‘stage of change’ for each risk factor, separately. The stages of change were determined by the question “Which statement fits best for you?”, where each answer option reflects one of the following stages of change: pre-contemplation, contemplation, preparation, action or maintenance [11]. Subsequently, participants were divided into two groups based on the stages of change. Participants who had the *intention to change* had indicated that they are in the contemplation (i.e., aware of unhealthy behaviour and planning to change in the coming 6 months) or the preparation (i.e., planning preparatory actions for health behaviour change in the next 30 days) stage. Participants who had *no intention to change* had indicated to be in the pre-contemplation (i.e., no intention to change health behaviour), action (i.e., changed health behaviour in the last 6 months) or maintenance (i.e., maintained improved health behaviour for more than 6 months) stage. This classification was made since participants could indicate that they are in the maintenance or action group, despite still not adhering to a healthy lifestyle according to the guidelines [23–25].

A detailed description of the measurements of the four behavioural-related risk factors for dementia (i.e., physical inactivity, poor-to-moderate adherence to a Mediterranean diet, excessive alcohol consumption and smoking) can be found in Additional file 1: Appendix 2. Briefly, physical inactivity is defined as less than 150 min moderate to vigorous physical activity per week and less than two times per week doing strength exercises [24]. The adherence to the Mediterranean Intervention for Neurodegenerative Delay (MIND) diet was determined based on the intake of nine food components of the MIND diet (e.g. legumes, vegetables, fruit, fish, meat, poultry, nuts, cheese and olive oil) [25, 26]. Alcohol consumption was measured using the following two questions “How often did you drink alcohol in the past month?” and “How many glasses did you drink on average per day?”. Subsequently, the number of glasses of alcohol per week was calculated in order to classify participants into: 1) non-alcohol consumers, 2) low/moderate alcohol consumers or 3) excessive alcohol consumers. Excessive alcohol consumers are defined as people who drink on average more than one glass of alcohol per day or binge drink (more than three glasses alcohol per occasion for females and more than four glasses alcohol per occasion for males) [23]. Further, smoking behaviour was assessed with the following two questions: “Did you smoke in the past month?” and “Have you ever smoked a full year?”. Non-smokers are defined as people who never smoked for more than a year and also did not smoke in the past month. Current smokers are defined as people who reported smoking in the past month [27]. Ex-smokers are defined as people who reported smoking for more than 1 year in the past, but did not smoke in the past month.

#### Covariates

Age (in years) was included as a categorical variable in the analyses (30 to 45 years (young individuals), 45 to 65 years (middle-aged individuals) and 65 to 80 years (older individuals)). Sex was included as a dichotomous variable (male/female). Further, education is based on the question “What is your highest level of education?” [28]. Due to underrepresentation of lower education groups, highest level of education is used as a dichotomous variable (low to middle /high). Finally, employment (working for at least 1 hour per week) is included as a dichotomous variable (yes/no) [29].

### Statistical analyses

The characteristics of the participants, including socio-demographic variables, knowledge about dementia risk reduction, the MCLHB-DRR subscale scores, health behaviour status and the intention to change health behaviours were explored using descriptive statistics. Differences between demographic subgroups on the MCLHB-DRR subscale scores and dementia knowledge statements were estimated using independent t-tests (normally distributed continuous variables; two groups), one-way ANOVA (normally distributed continuous variables; three or more groups) and Chi-squared tests (categorical variables) were used. A *p*-value < 0.05 was considered to be significant. Univariable (model 0) and multivariable logistic regression analyses were conducted, between the MCLHB-DRR subscale scores and the intention to change health behaviours (model 1), and subsequently adjusted for potential confounders age, sex, education and employment (model 2) among participants with unhealthy behaviours. Additionally, interaction effects between MCLHB-DRR subscale scores and the potential confounders were assessed. The associations were stratified if any interaction term was significant (*p* < 0.05) (model 3). Statistical analyses were performed using SPSS version 21 for Windows [30].

## Results

### Characteristics of the study population

From the 4500 selected eligible participants, 658 participants completed the survey, which resulted in a response rate of 15%. Three participants were excluded for erroneous outliners. After exclusion of these participants, the data of 655 participants were left for analysis (see the flowchart of participants recruitment in Additional file 1: Appendix 3). The characteristics of the total study population are presented in Table 1.

**Table 1:** Characteristics of the total study population*

Characteristics	Total study population (*n* = 655)
**Age in years, mean (SD)**	57.6 (13.4)
30–45 years	137 (21%)
45–65 years	303 (46%)
65–80 years	215 (33%)
**Gender, female**	355 (54%)
**Partner, yes**	488 (75%)
**Education**	
Low to middle	262 (40%)
High	393 (60%)
**Employed, yes**	371 (57%)
Work hours, mean (SD)	31.8 (11.7)
**Health behaviours**	
Physically inactive	391 (60%)
Poor-to-moderate adherence to MIND diet	655 (100%)
Excessive alcohol user	222 (34%)
Current smoker	82 (13%)

*Abbreviations*: *N* number, *SD* standard deviation; *Noted in *N* (%) unless indicated otherwise

### Knowledge about dementia and dementia risk reduction

In general, participants were not aware or had incorrect knowledge about dementia (risk reduction) (see Table 2). The majority of the participants (67.6%) believed that dementia is a normal part of the ageing process. Although the majority of the participants (62.3%) were aware of the possibility to reduce the risk of developing dementia by maintaining a healthy lifestyle, only 31.1% of the participants indicated high blood pressure as a risk factor for dementia. Moreover, 25.0% of the participants did not know whether it is possible to reduce the risk of developing dementia. Overall, highly educated participants were better informed about the risk factors for dementia (37% vs. 22%) and possibility for dementia risk reduction (68% vs. 54%), compared to low to middle educated participants. Older participants (16.8%) more often incorrectly believed that it is not possible to reduce the risk of developing dementia compared to the younger participants (5.4%).

**Table 2:** Differences in knowledge about dementia risk reduction between demographic subgroups*

	Dementia is a normal part of the ageing process [FALSE]				Maintaining a healthy lifestyle reduces the risk of developing the most common forms of dementia [TRUE]				Having high blood pressure increases a person’s risk of developing dementia [TRUE]			
	False	True	I don’t know	***P*** value	False	True	I don’t know	***P*** value	False	True	I don’t know	***P*** value
**Total study population**	179 (27.3%)	443 (67.6%)	33 (5.0%)		83 (12.7%)	408 (62.3%)	164 (25.0%)		91 (13.9%)	204 (31.1%)	360 (55.0%)	
**Age**				0.535				0.029				0.106
30–45 years	36 (27.9%)	86 (66.7%)	7 (5.4%)		7 (5.4%)	83 (64.3%)	39 (30.2%)		9 (7.0%)	44 (34.1%)	76 (58.9%)	
45–65 years	71 (25.2%)	200 (70.9%)	11 (3.9%)		35 (12.4%)	178 (63.1%)	69 (24.5%)		40 (14.2%)	85 (30.1%)	157 (55.7%)	
65–80 years	72 (29.5%)	157 (64.3%)	15 (6.1%)		41 (16.8%)	147 (60.2%)	56 (23.0%)		42 (17.2%)	75 (30.7%)	127 (52.0%)	
**Sex**				0.000				0.119				0.530
Male	106 (35.3%)	178 (59.3%)	16 (5.3%)		38 (12.7%)	198 (66.0%)	64 (21.3%)		45 (15.0%)	97 (32.3%)	158 (52.7%)	
Female	73 (20.6%)	265 (74.6%)	17 (4.8%)		45 (12.7%)	210 (59.2%)	100 (28.2%)		46 (13.0%)	107 (30.1%)	202 (56.9%)	
**Education**				0.446				0.001				0.000
Low to middle	78 (30.1%)	167 (64.5%)	14 (5.4%)		44 (17.0%)	141 (54.4%)	74 (28.6%)		48 (18.5%)	56 (21.6%)	155 (59.8%)	
High	101 (25.9%)	270 (69.2%)	19 (4.9%)		37 (9.5%)	264 (67.7%)	89 (22.8%)		41 (10.5%)	146 (37.4%)	203 (52.1%)	
**Employment**				0.141				0.361				0.194
Unemployed	82 (28.9%)	183 (64.4%)	19 (6.7%)		42 (14.8)	173 (60.9%)	69 (24.3%)		43 (15.1%)	78 (27.5%)	163 (57.4%)	
Employed	97 (26.1%)	260 (70.1%)	14 (3.8%)		41 (11.1%)	235 (63.3%)	95 (25.6%)		48 (12.9%)	126 (34.0%)	197 (53.1%)	

*Values presented in *N* (%)

### Health beliefs and attitudes towards dementia and dementia risk reduction

**Fig. 1:**
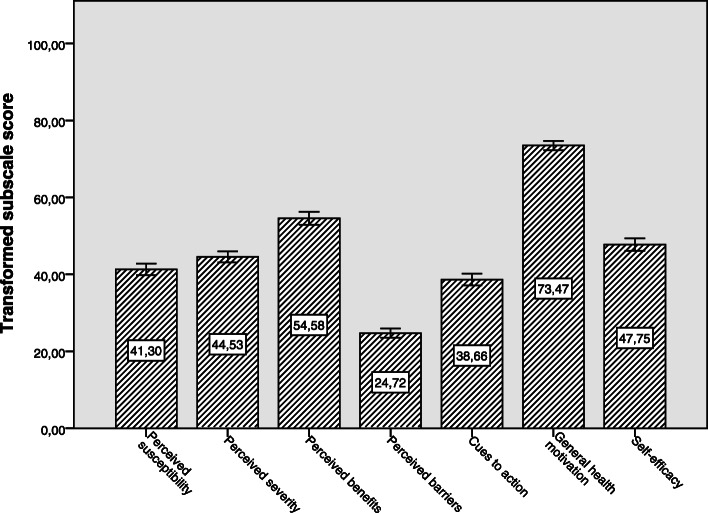
Bar chart with 95%-confidence intervals of the transformed MCLHB-DRR subscale scores on a 100-point scale for the total study population

The MCLHB-DRR subscale scores are presented in Table 3. The transformed subscale scores are presented in Fig. 1. In general, the study population had relatively high general health motivation and perceived benefit scores. However, relatively low perceived susceptibility, perceived severity, cues to action and self-efficacy scores (< 50 points on a 100-point scale) were found. Older, female and unemployed participants had higher scores on perceived severity compared to the younger, male and employed participants. Whereas, the younger, highly educated and employed participants had higher scores on perceived benefits and self-efficacy. The younger and low to middle educated participants also perceived more barriers compared to older and highly educated participants.

**Table 3:** MCLHB-DRR subscale scores for different demographic subgroups*

Characteristics	Perceived susceptibility (range: 3–15)	***p***-value	Perceived severity (range: 5–25)	***p***-value	Perceived benefits (range: 2–10)	***p***-value	Perceived barriers (range: 4–20)	***p***-value	Cues to action (range: 4–20)	***p***-value	General health motivation (range: 3–15)	***p***-value	Self-efficacy (range: 2–10)	***p***-value
**Total study population**	8.0 (2.3)		13.9 (3.7)		6.4 (1.8)		8.0 (2.5)		10.2 (3.1)		11.8 (1.9)		5.8 (1.7)	
**Age**		0.095		0.001		0.000		0.041		0.642		0.054		0.000
30–45 years	8.3 (2.4)		13.0 (3.6)		7.0 (1.7)		8.4 (2.7)		10.2 (3.4)		11.5 (1.8)		6.4 (1.6)	
45–65 years	8.0 (2.4)		13.9 (3.6)		6.3 (1.8)		7.9 (2.4)		10.3 (3.3)		11.8 (1.8)		5.8 (1.7)	
65–80 years	7.7 (2.2)		14.5 (3.8)		6.0 (1.7)		7.7 (2.4)		10.0 (2.7)		12.0 (2.0)		5.4 (1.5)	
**Sex**		0.068		0.022		0.657		0.603		0.520		0.283		0.382
Male	7.8 (2.4)		13.5 (3.8)		6.4 (1.9)		7.9 (2.5)		10.1 (3.2)		11.7 (1.9)		5.9 (1.9)	
Female	8.1 (2.2)		14.2 (3.6)		6.3 (1.7)		8.0 (2.6)		10.3 (3.1)		11.9 (1.9)		5.8 (1.5)	
**Education**		0.525		0.827		0.023		0.008		0.804		0.356		0.000
Low to middle	8.0 (2.3)		13.9 (3.9)		6.2 (1.8)		8.3 (2.4)		10.1 (3.0)		11.9 (1.9)		5.5 (1.6)	
High	7.9 (2.3)		13.9 (3.6)		6.5 (1.7)		7.7 (2.6)		10.2 (3.2)		11.8 (1.9)		6.0 (1.7)	
**Employment**^**†**^		0.824		0.013		0.048		0.241		0.897		0.253		0.002
Unemployed	8.0 (2.3)		14.3 (3.7)		6.2 (1.7)		7.8 (2.4)		10.2 (2.9)		11.9 (2.0)		5.6 (1.6)	
Employed	7.9 (2.4)		13.6 (3.7)		6.5 (1.8)		8.1 (2.6)		10.2 (3.3)		11.7 (1.8)		6.0 (1.7)	

*Abbreviations*: *MCLHB-DRR* Motivation to Change Lifestyle and Health Behaviour for Dementia Risk Reduction. *Values presented in mean (SD) unless indicated otherwise

### The intention to change health behaviours

Characteristics of the subpopulations with unhealthy behaviours (i.e., physical inactivity, poor-to-moderate adherence to the MIND diet, excessive alcohol consumption and smoking) are presented in Table 4. More than half of the participants (60%) were physically inactive, and only 32% of them had the intention to change their physical activity. None of the participants completely adhered to the MIND-diet and only 18% had the intention to change their diet. Further, 34% of the participants were excessive alcohol consumers of whom 29% had the intention to change their alcohol consumption. Finally, 13% of the participants were currently smoking and the majority (59%) of the smokers had the intention to change their smoking behaviour. The characteristics of participants stratified by health behaviour status are presented in Additional file 1: Appendix 4.

**Table 4:** Characteristics of the four sub-populations with poor lifestyle habits stratified for the intention to change health behaviours*

	Physical inactivity (*N* = 391)		Poor-to-moderate adherence to MIND diet (*N* = 655)		Excessive alcohol consumption (*N* = 222)		Smoking (*N* = 82)	
	No intention to change	Intention to change	No intention to change	Intention to change	No intention to change	Intention to change	No intention to change	Intention to change
*N*	264 (68%)	127 (32%)	535 (82%)	120 (18%)	157 (71%)	65 (29%)	34 (41%)	48 (59%)
Age in years, mean (SD)	59.8 (12.3)	54.8 (13.3)	58.6 (13.1)	52.8 (13.7)	60.9 (11.7)	54.7 (13.5)	59.7 (11.5)	52.4 (12.1)
Age								
30–45 years	40 (53%)	36 (47%)	99 (72%)	38 (28%)	18 (49%)	19 (51%)	4 (21%)	15 (79%)
45–65 years	126 (66%)	64 (34%)	244 (81%)	59 (19%)	75 (72%)	29 (28%)	18 (40%)	27 (60%)
65–80 years	98 (78%)	27 (22%)	192 (89%)	23 (11%)	64 (79%)	17 (21%)	12 (67%)	6 (33%)
Sex, female	135 (64%)	75 (36%)	283 (80%)	72 (20%)	56 (65%)	30 (35%)	14 (31%)	31 (69%)
Education								
Low to middle	120 (69%)	53 (31%)	206 (79%)	56 (21%)	60 (82%)	13 (18%)	18 (44%)	23 (56%)
High	144 (66%)	74 (34%)	329 (61%)	64 (39%)	97 (65%)	52 (35%)	16 (39%)	25 (61%)
Employment, yes	143 (62%)	87 (38%)	287 (77%)	84 (23%)	79 (65%)	43 (35%)	21 (39%)	33 (61%)
Physically inactive	–	–	311 (80%)	80 (20%)	87 (71%)	36 (29%)	24 (44%)	31 (56%)
Excessive alcohol consumption	86 (70%)	37 (30%)	181 (82%)	41 (18%)	–	–	19 (46%)	22 (54%)
Smoking	42 (76%)	13 (24%)	66 (80%)	16 (20%)	28 (68%)	13 (32%)	–	–

*Abbreviations*: *SD* standard deviation, *N* number; *Values presented in *N* (%), unless indicated otherwise

In Table 5, the results of the final multivariable regression analyses investigating the association between health beliefs and attitudes towards dementia (risk reduction) and the intention to change health behaviours among participants with unhealthy behaviours are presented (see Additional file 1: Appendix 5 for the results of all analyses, including univariable regression analyses). Physically inactive individuals who had relatively high perceived benefits (OR = 1.33, 95%-CI:1.11–1.58) or cues to action scores (OR = 1.13, 95%-CI:1.03–1.24) had more often the intention to change physical activity, than those who had lower scores for perceived benefits or cues to action. Individuals with high perceived barriers scores had more often the intention to change their diet (OR = 1.10, 95%-CI:1.01–1.21). In addition, less educated individuals with high perceived benefits scores had less often the intention to change their diet (OR = 0.78, 95%-CI:0.60–0.99), while higher educated individuals with high perceived benefits scores had more often the intention to change their diet (OR = 1.41, 95%-CI:1.12–1.78). Excessive alcohol consumers with relatively high perceived benefits (OR = 1.30, 95%-CI:1.00–1.69) or cues to action scores (OR = 1.17, 95%-CI:1.02–1.35), had more often the intention to change their alcohol consumption behaviour. Younger excessive alcohol consumers with a relatively high perceived severity scores had more often the intention to change their alcohol consumption behaviour (OR = 2.70, 95%-CI:1.04–6.97), while the opposite is true for middle-aged excessive alcohol consumers (OR = 0.81, 95%-CI:0.67–0.99). Finally, smokers who perceived more barriers had less often the intention to change their smoking behaviour (OR = 0.78, 95%-CI:0.63–0.98). Overall, older individuals were less likely to change their health behaviour.

**Table 5:** The results of the multivariable logistic regression analyses investigating the association between MCLHB-DRR subscales and the intention to change health behaviours^#^

	Intention to change physical activity(***n*** = 391)	Intention to change diet (***n*** = 655)	Intention to change alcohol consumption (***n*** = 222)	Intention to change smoking behaviour (***n*** = 82)
**Independent variables**	OR (95%-CI)	OR (95%-CI)	OR (95%-CI)	OR (95%-CI)
Perceived susceptibility	0.96 (0.87;1.07)	1.00 (0.91;1.11)	0.97 (0.84;1.14)	1.08 (0.84;1.39)
Perceived severity	1.00 (0.94;1.07)	1.04 (0.97;1.10)	0.97 (0.87;1.07)*	1.05 (0.89;1.24)
Perceived benefits	**1.33 (1.11;1.58)**	1.07 (0.91;1.26)*	**1.30 (1.00;1.69)**	0.88 (0.58;1.34)
Perceived barriers	1.03 (0.93;1.13)	**1.10 (1.01;1.21)**	1.00 (0.86;1.15)	**0.78 (0.63;0.98)**
Cues to action	**1.13 (1.03;1.24)**	1.06 (0.98;1.16)	**1.17 (1.02;1.35)**	1.19 (0.93;1.52)
General health motivation	1.14 (0.99;1.30)	0.97 (0.98;1.16)	1.05 (0.86;1.27)	1.01 (0.78;1.33)
Self-efficacy	0.93 (0.78;1.10)	1.05 (0.89;1.24)	0.88 (0.68;1.14)	0.89 (0.54;1.46)
Female (ref: male)	1.24 (0.77;1.98)	1.23 (0.80;1.88)	1.59 (0.83;3.06)	**3.95 (1.14;13.73)**
Age, 45–65 years (ref: 30–45 years)	0.55 (0.30;1.00)	0.66 (0.40;1.10)	0.46 (0.19;1.08)	**0.19 (0.04;0.97)**
Age, 65–80 years (ref: 30–45 years)	0.38 (0.16;0.91)	**0.45 (0.21;0.97)**	0.40 (0.12;1.34)	**0.05 (0.01;0.40)**
Education high (ref: education low-middle)	0.97 (0.60;1.58)	**0.56 (0.36;0.87)**	**2.23 (1.03;4.84)**	1.54 (0.48;4.91)
Employed (ref: unemployed)	1.27 (0.67;2.41)	1.60 (0.90;2.84)	1.16 (0.46;2.96)	1.05 (0.30;3.73)

*Abbreviations*: *OR* odds ratio, *CI* confidence interval; ^#^ adjusted for age, sex, educational level, employment and other MCLHB-DRR subscales

*see Additional file 1: Appendix 5 for the stratified results, due to a significant interaction term

## Discussion

This study shows that the knowledge about dementia (risk reduction) is poor among the Dutch general population. In addition, older participants perceived dementia as a more severe disease compared to younger participants, but they perceived less benefits and barriers of performing health-enhancing behaviour for dementia risk reduction and had less confidence in their ability to perform the desired behaviour. Highly educated participants perceived less barriers and more benefits, but also had more confidence in their ability to perform the desired behaviour compared to less educated participants. Furthermore, a large proportion of the participants had an unhealthy behaviour, of which only a small proportion had the intention to change health behaviour. Perceived benefits and cues to action were associated with the intention to change physical activity and alcohol consumption. Among younger excessive alcohol consumers, also perceived severity was associated with the intention to change alcohol consumption. Perceived barriers were associated with the intention to change diet. Among highly educated participants, also perceived benefits were associated with the intention to change diet, but inversely associated among the less educated participants. Smokers who perceived more barriers to change their smoking behaviour were less likely to have the intention to change this behaviour.

### Knowledge about dementia and dementia risk reduction

A large proportion of the participants was unaware or had insufficient knowledge about dementia (risk reduction), especially older and less educated individuals. For instance, the majority (62%) of the participants had the misconception that dementia is a normal part of the ageing process. This percentage is slightly higher compared to the findings of previous studies over the world, where nearly half of the participants (median 48%, range 39–75%; 13 studies) believed that dementia is a normal part of ageing [6]. Further, although 68% of the participants were aware of the possibility to reduce dementia risk by maintaining a healthy lifestyle, still a considerable proportion of the participants (25%) did not know whether it is possible to reduce dementia risk and only around a third (31%) of the participants indicated high blood pressure as a risk factor for dementia. These findings are quite similar to the findings of a recent survey conducted in the Netherlands [7].

### Health beliefs and attitudes towards dementia and dementia risk reduction

Older participants perceived dementia as a more severe disease compared to younger participants. This can be explained by the fact that dementia incidence increases with age and were therefore older individuals are more likely to know someone with dementia. On the other hand, older participants perceived less benefits, and barriers of performing health-enhancing behaviours and had less confidence in their ability to perform the desired behaviour compared to younger participants. This could suggest that older individuals may think that they benefit less from behavioural changes or do not benefit at all, reflecting the misconception that dementia is an inevitable age-related disease for which health behaviour changes might not be effective anymore to prevent of postpone cognitive decline. Further, highly educated participants perceived more benefits and less barriers to perform healthy behaviours and had more confidence in their ability to perform the desired behaviour. These findings are in line with previous findings [15, 20]. Only two previous studies reported MCLHB-DRR subscale scores reflecting the health beliefs and attitudes towards dementia (risk reduction) among the Australian (50 years and older) and Turkish (40 years and older) population [14, 15]. In comparison to our study, these studies showed slightly higher scores on a number of subscales of the MCLHB-DRR scale. However, these differences in subscale scores are relatively small when taking into account the different scoring possibilities in the Australian, Turkish and Dutch version of the MCLHB-DRR (see Additional file 1: Appendix 6). Furthermore, similarly to our study, they also found relatively high scores on perceived benefits and general health motivation.

### Intention to change health behaviours

Among participants with unhealthy behaviours, perceived benefits and cues to action were associated with the intention to change physical activity and alcohol consumption, and perceived barriers were associated with the intention to change diet and inversely associated with the intention to change smoking behaviour. Moreover, perceived severity was associated with the intention to change alcohol consumption among younger individuals and perceived benefits was associated with the intention to change diet among higher educated individuals. These findings suggest that providing information about dementia symptoms and the benefits of health behaviour change for dementia risk reduction may enlarge the intention to change physical activity and alcohol consumption. In case of diet, we found that having more barriers could lead to the intention to change diet for reducing dementia risk. This is not what we would expect. However, this may also reflect that people are having problems (barriers) with changing their diet. A previous study has shown that healthy eating comes with a lot of barriers, such as time and taste related factors [31]. Therefore, individuals who are taking preparatory actions in order to improve their diet might experience more barriers compared to individuals who do not have the intention to change their diet. Among higher educated individuals, we found that perceiving more benefits of changing lifestyle for dementia risk reduction could lead to the intention to change diet, while the opposite is true for lower educated individuals. These results could indicate that lower educated individuals might think that they have a healthy diet and do not need to change their diet. Therefore, education about a healthy diet is important, especially among the lower educated individuals. Further, we found that having less barriers could lead to the intention to change smoking behaviour for reducing dementia risk. Therefore, interventions to change smoking behaviour should focus more on lowering the barriers to enhance the intention to change smoking behaviour. More research is needed to get insight in the specific barriers for changing smoking behaviour. In general, our findings are consistent with previous studies [16, 17].

### Strengths and limitations

This is the first study that investigated the health beliefs and attitudes towards dementia (risk reduction) in the Dutch general population. A major strength of this study was the stratified random sample and its adequate sample size of 655 participants. This study had, however, certain limitations. First, the response rate was relatively low (17%), despite several attempts to increase the response rate (i.e., an easily accessible link to the survey, lottery to win a voucher and an offer to receive the findings of the survey). Furthermore, 60% of our study population consisted of highly educated individuals, which is a representative sample of the municipality of Groningen, but not for the Dutch general population. Further, it might not be clear to which behavioural changes participants were referring to when completing the MCLHB-DRR questionnaire. For instance, with the statement ‘Changing my lifestyle and health habits can help me reduce my chance of developing dementia’, participants could refer to a specific health behaviour, for example smoking or physical activity. Participants possibly did not even know whether and which health behaviours are important risk factors for dementia.

### Implications

The findings of this study indicate that individuals’ knowledge, health beliefs and attitudes towards dementia (risk reduction) need to be improved, which can be done in several ways. First, especially younger individuals should become more aware of the symptoms and severity of dementia. For example, by creating a more dementia friendly society in which lessons are given on what dementia is, what difficulties patients with dementia may experience and how this affects their families. This may help younger individuals to acknowledge the importance of a healthy lifestyle for reducing the risk of developing dementia later in life. Second, the perceived benefits of health behaviour change should be emphasized, especially among older and less educated individuals. This may help to motivate these individuals to adopt a healthier lifestyle in order to reduce their dementia risk. Finally, further research should explore the perceived barriers to change their smoking behaviour and diet and the cues to action to change their physical activity and alcohol consumption.

## Conclusions

This study shows that the knowledge, health beliefs and attitudes towards dementia and dementia risk reduction among the Dutch general population is not sufficient to support dementia risk reduction. More education about dementia and dementia risk reduction is needed to improve the knowledge, health beliefs and attitudes towards dementia and dementia risk reduction in order to change health behaviour. Future research should investigate the effectiveness of dementia prevention campaigns aimed to improve the knowledge, beliefs and attitudes toward dementia (risk reduction) and the intention and actual change of health behaviours.

## Supplementary Information

**Additional file 1: Appendix 1.** Validation of the Dutch version of the DKAS scale. **Appendix 2.** Measurements of lifestyle related risk factors of dementia. **Appendix 3.** Flowchart of potential participants selected and stratified by age and sex. **Appendix 4.** Characteristics of participants stratified by health behaviour status. **Appendix 5.** Summary of the results of the univariable and multivariable regression analyses. **Appendix 6.** Comparison of MCLHB-DRR scale scores.

## Data Availability

The data collected during this study will be available from the corresponding author upon reasonable request.

## Abbreviations

HBM: Health Belief Model
MCLHB-DRR: Motivation to Change Lifestyle and Health Behaviours for Dementia Risk Reduction
DKAS: Dementia Knowledge Assessment Scale
MIND: diet Mediterranean Intervention for Neurodegenerative Delay diet
OR: Odds Ratio

## References

[1] World Health Organization. Dementia [Internet]. 2019. Available from: https://www.who.int/news-room/fact-sheets/detail/dementia

[2] Alzheimer Nederland. Factsheet: cijfers en feiten over dementie; 2019 [Internet]. [cited 2020 Oct 19]. Available from: https://www.alzheimer-nederland.nl/factsheet-cijfers-en-feiten-over-dementie.

[3] World Health Organization (WHO). Towards a dementia plan: a WHO guide. [Internet]. 2018. Available from: https://www.who.int/health-topics/dementia#tab=tab_1

[4] Livingston G, Sommerlad A, Orgeta V, Costafreda SG, Huntley J, Ames D, Ballard C, Banerjee S, Burns A, Cohen-Mansfield J, Cooper C, Fox N, Gitlin LN, Howard R, Kales HC, Larson EB, Ritchie K, Rockwood K, Sampson EL, Samus Q, Schneider LS, Selbæk G, Teri L, Mukadam N (2017). The lancet commissions dementia prevention, intervention, and care. Lancet..

[5] Livingston G, Huntley J, Sommerlad A, Ames D, Ballard C, Banerjee S, Brayne C, Burns A, Cohen-Mansfield J, Cooper C, Costafreda SG, Dias A, Fox N, Gitlin LN, Howard R, Kales HC, Kivimäki M, Larson EB, Ogunniyi A, Orgeta V, Ritchie K, Rockwood K, Sampson EL, Samus Q, Schneider LS, Selbæk G, Teri L, Mukadam N (2020). Dementia prevention, intervention, and care: 2020 report of the lancet commission. Lancet..

[6] Cations M, Radisic G, Crotty M, Laver KE. What does the general public understand about prevention and treatment of dementia? A systematic review of population-based surveys. PLoS One. 2018;13(4):e0196085. 10.1371/journal.pone.0196085 PMC5908164 29672559

[7] Heger I, Deckers K, van Boxtel M, de Vugt M, Hajema K, Verhey F, Köhler S (2019). Dementia awareness and risk perception in middle-aged and older individuals: baseline results of the MijnBreincoach survey on the association between lifestyle and brain health. BMC Public Health.

[8] Cahill S, Pierce M, Werner P, Darley A, Bobersky A (2015). A systematic review of the public’s knowledge and understanding of Alzheimer’s disease and dementia. Alzheimer Dis Assoc Disord.

[9] Kelly S, Martin S, Kuhn I, Cowan A, Brayne C, Lafortune L (2016). Barriers and Facilitators to the Uptake and Maintenance of Healthy Behaviours by People at Mid-Life: A Rapid Systematic Review. Wang Y, editor. PLoS One.

[10] Janz NK, Becker MH. The health belief model: a decade later. Heal Educ Behav. 1984;11(1):1–47. 10.1177/109019818401100101 6392204

[11] Prochaska JO, Velicer WF (1997). The transtheoretical model of health behavior change. Am J Health Promot.

[12] De Vries H, Mesters I, Van’t Riet J, Willems K, Reubsaet A (2006). Motives of Belgian adolescents for using sunscreen: the role of action plans. Cancer Epidemiol Biomark Prev.

[13] de Vries H. An Integrated Approach for Understanding Health Behavior; The I-Change Model as an Example. Psychol Behav Sci Int J. 2017:2.

[14] Kim S, Sargent-Cox K, Cherbuin N, Anstey KJ (2014). Development of the motivation to change lifestyle and health behaviours for dementia risk reduction scale. Dement Geriatr Cogn Dis Extra.

[15] Akyol MA, Zehirlioğlu L, Erünal M, Mert H, Hatipoğlu NŞ, Küçükgüçlü Ö (2020). Determining Middle-Aged and Older Adults’ Health Beliefs to Change Lifestyle and Health Behavior for Dementia Risk Reduction. Am J Alzheimer’s Dis Dementias®.

[16] Seifan A, Ganzer CA, Vermeylen F, Parry S, Zhu J, Lyons A (2017). Development and validation of the Alzheimer’s prevention beliefs measure in a multi-ethnic cohort—a behavioral theory approach. J Public Heal (United Kingdom).

[17] Smith BJ, Ali S, Quach H (2015). The motivation and actions of Australians concerning brain health and dementia risk reduction. Heal Promot J Aust.

[18] Annear MJ, Toye C, Elliott KEJ, McInerney F, Eccleston C, Robinson A (2017). Dementia knowledge assessment scale (DKAS): confirmatory factor analysis and comparative subscale scores among an international cohort. BMC Geriatr.

[19] Beaton DE, Bombardier C, Guillemin F, Ferraz MB (2000). Guidelines for the process of cross-cultural adaptation of self-report measures. Spine (Phila Pa 1976).

[20] Smith BJ, Ali S, Quach H (2014). Public knowledge and beliefs about dementia risk reduction: a national survey of Australians. BMC Public Health.

[21] Low LF, Anstey KJ (2009). Dementia literacy: recognition and beliefs on dementia of the Australian public. Alzheimers Dement.

[22] Joxhorst T, Vrijsen J, Niebuur J, Smidt N (2020). Cross-cultural validation of the motivation to change lifestyle and health behaviours for dementia risk reduction scale in the Dutch general population. BMC Public Health.

[23] Kromhout D, Spaaij CJK, De Goede J, Weggemans RM, Brug J, Geleijnse JM (2016). The 2015 Dutch food-based dietary guidelines. Eur J Clin Nutr.

[24] Weggemans RM, Backx FJG, Borghouts L, Chinapaw M, Hopman MTE, Koster A (2018). The 2017 Dutch physical activity guidelines. Int J Behav Nutr Phys Act.

[25] Morris MC, Tangney CC, Wang Y, Sacks FM, Bennett DA, Aggarwal NT (2015). MIND diet associated with reduced incidence of Alzheimer’s disease. Alzheimers Dement.

[26] Morris MC, Tangney CC, Wang Y, Sacks FM, Barnes LL, Bennett DA, Aggarwal NT (2015). MIND diet slows cognitive decline with aging. Alzheimers Dement.

[27] Slagter SN, Van Vliet-Ostaptchouk JV, Vonk JM, Boezen HM, Dullaart RPF, Muller Kobold AC, et al. Combined effects of smoking and alcohol on metabolic syndrome: The lifelines cohort study. PLoS One. 2014;9(4). 10.1371/journal.pone.0096406 PMC4004580 24781037

[28] Klijs B, Kibele EUB, Ellwardt L, Zuidersma M, Stolk RP, Wittek RPM, Mendes de Leon CM, Smidt N (2016). Neighborhood income and major depressive disorder in a large Dutch population: results from the LifeLines cohort study. BMC Public Health.

[29] International Labour Organization. International Training Compendium on Labour Statistics. 2003.

[30] IBM Corp. IBM SPSS statistics for windows, version 21.0. Armonk: IBM Corp; 2012.

[31] Kearney JM, McElhone S (1999). Perceived barriers in trying to eat healthier - results of a pan-EU consumer attitudinal survey. Br J Nutr.

