# Supplementary material for: Knowledge, health beliefs and attitudes towards dementia and dementia risk reduction among the Dutch general population: a cross-sectional study
Source: BMC Public Health. 2021 May 3;21:857. doi: 10.1186/s12889-021-10913-7 (PMC8094456; doi:10.1186/s12889-021-10913-7)
Supplement: Supplementary file 1 — Additional file 1: Appendix 1. Validation of the Dutch version of the DKAS scale. Appendix 2. Measurements of lifestyle related risk factors of dementia. Appendix 3. Flowchart of potential participants selected and stratified by age and sex. Appendix 4. Characteristics of participants stratified by health behaviour status. Appendix 5. Summary of the results of the univariable and multivariable regression analyses. Appendix 6. Comparison of MCLHB-DRR scale scores. [file 12889_2021_10913_MOESM1_ESM.docx]

**TITLE:** Knowledge, health beliefs and attitudes towards dementia and dementia risk reduction and among the Dutch general population: a cross-sectional study

**AUTHORS**

J. Vrijsen^1^*, T.F. Matulessij^1^, T. Joxhorst^1^, S.E. de Rooij^2^, N. Smidt^1^

^1^ University of Groningen, University Medical Centre Groningen, Department of Epidemiology, Groningen, the Netherlands

^2^ University of Groningen, University Medical Centre Groningen, Department of Internal Medicine, Groningen, the Netherlands

***Corresponding author** University of Groningen, University Medical Centre Groningen, Department of Epidemiology, Hanzeplein 1, PO Box 30 001, FA40, 9700 RB Groningen, the Netherlands.

E-mail: j.vrijsen@umcg.nl; Phone: +31625650782

**Appendices**

Appendix 1: Validation of the Dutch version of the DKAS scale

Appendix 2: Measurements of lifestyle related risk factors of dementia

Appendix 3: Flowchart of potential participants selected and stratified by age and sex

Appendix 4: Characteristics of participants stratified by health behaviour status

Appendix 5: Summary of the results of the univariable and multivariable regression analyses

Appendix 6: Comparison of MCLHB-DRR scale scores

**Appendix 1*.*** Results of the cross-cultural validation of the Dutch version of the DKAS scale

**Background**

The Dementia Knowledge Assessment Scale (DKAS) was developed in Australia and measures dementia knowledge in diverse populations, including the general population (Annear et al. 2017). However, there is currently no instrument available to measure dementia knowledge among the Dutch general population. Therefore, we aimed to translate and validate the Dementia Knowledge Assessment Scale (DKAS).

**Methods**

**Questionnaire**

The DKAS consists of 25 statements about dementia that are factually correct or incorrect, based on a literature review and international Delphi study with experts on dementia. This scale is capable of elucidating knowledge characteristics across four coherent domains: a) Causes and Characteristics, b) Communication and Behaviour, c) Care Considerations, and d) Risks and Health Promotion. Respondents could answer each item on a 5-point Likert scale with five response options (i.e., false, probably false, probably true, true and I don’t know) (Annear et al. 2017).

**Scale translation**

For the translation of the DKAS scale, we used the method of Beaton et al. (2000). Briefly, the DKAS was translated into Dutch by three native Dutch translators, independently. Two of these translators were familiar with the concepts being examined in the questionnaire (informed translators). The third translator was not familiar with the content or concepts of the questionnaire (uninformed translator). All items, instructions and the response options of the questionnaire were translated. Subsequently, the three translated versions were synthesized to one Dutch version. Secondly, the synthesized Dutch version of the questionnaire was translated back into English by two independent native English speakers (uninformed translators). Afterwards all versions of the questionnaire were discussed by the informed translators and consensus about the pre-final version of the questionnaire was reached. Finally, the two back translations were combined and this version was sent to the developers of the original scale to check whether the meaning of the translated items was equivalent to the meaning of the original items.

**Statistical analysis**

Exploratory factor analysis (EFA) was performed. Principal Axis Factoring was used, since the data was not normally distributed. Oblique rotation was used as a rotation method, which takes into account the correlations among factors. If correlations between factors were below 0.32, we changed to Varimax rotation. Items that did not have a correlation of 0.20 or higher with any of the other items were deleted immediately. Items with a high correlation (> 0.70) with any of the other items, were considered carefully. Items with a factor loading below 0.30 on any of the factors were deleted immediately. Deletion of an item was considered if the item did not load sufficiently on one of the factors (<0.50) or if an item had a cross-loading greater than 0.30. Internal consistency of the subscales was evaluated by item-total correlations and Cronbach’s alpha. Deletion of an item was considered when the item-total correlation of an item was below 0.30. Cronbach’s alpha values of 0.70 or higher were considered acceptable.

**Results**

Six hundred fifty-five participants completed an online survey including the Dutch DKAS questionnaire. The results of the EFA and internal consistency are shown in Table 1 and Table 2 below. The four-factor solution was evaluated as the original DKAS scale consists of four subscales. Item 4, 6 and 9 had an inter-item correlation smaller than 0.20 with all other items. All other inter-item correlations did not exceed 0.70. The Bartlett’s test of sphericity was significant, indicating that the data was adequate for factor analysis (p<0.001). The first four factors had eigenvalues greater than 1.00 and the scree plot also suggested a four-factor model. EFA showed many low item loadings and a small number of items loaded on their intended subscales. Item 4 and 23 had an item-total correlation below 0.30. Cronbach’s alpha values were α = 0.49 for Causes and Characteristics, α = 0.53 for Communication and Behaviour, α = 0.62 for Risk Factors and Health Promotion, α = 0.75 for Care Considerations, indicating poor internal consistency. Cronbach’s alpha of Causes and Characteristics, Risk Factors and Health Promotion and Care Considerations subscales could be elevated by deleting one or more items. EFA and internal consistency suggested deletion of the majority of the items. However, even with item reduction, three or four factors could not reach the threshold of a Cronbach’s alpha of 0.70 and would leave too little items left per factor.

**Table 1**: Exploratory factor analysis of the DKAS scale (N=655)*

|  | **Factor 1** | **Factor 2** | **Factor 3** | **Factor 4** |
| --- | --- | --- | --- | --- |
| Q1 Dementia is a normal part of the ageing process. | 0.20 |  |  |  |
| Q2 Alzheimer’s disease is the most common form of dementia. |  |  |  | **0.62** |
| Q3 People can recover from the most common forms of dementia. | 0.21 |  |  |  |
| Q4 Dementia does not result from physical changes in the brain. |  | 0.20 |  |  |
| Q5 Planning for end-of-life care is generally not necessary following a diagnosis of dementia. |  |  |  | 0.22 |
| Q6 Blood vessel disease (vascular dementia) is the most common form of dementia. |  |  |  |  |
| Q7 Most forms of dementia do not generally shorten a person’s life. |  |  |  | **0.46** |
| Q8 Having high blood pressure increases a person’s risk of developing dementia. |  | **0.56** |  |  |
| Q9 Maintaining a healthy lifestyle does not reduce the risk of developing the most common forms of dementia. |  | **0.50** |  |  |
| Q10 Symptoms of depression can be mistaken for symptoms of dementia. | 0.21 | 0.26 |  |  |
| Q11 Exercise is generally beneficial for people experiencing dementia. | 0.25 |  |  |  |
| Q12 Early diagnosis of dementia does not generally improve quality of life for people experiencing the condition. |  |  | -0.23 |  |
| Q13 The sudden onset of cognitive problems is characteristic of common forms of dementia. |  | **0.36** |  | 0.21 |
| Q14 It is impossible to communicate with a person who has advanced dementia. | 0.27 | 0.27 |  |  |
| Q15 A person experiencing advanced dementia will not generally respond to changes in their physical environment. | **0.68** |  |  |  |
| Q16 It is important to correct a person with dementia when they are confused. | **0.73** |  |  |  |
| Q17 People experiencing advanced dementia often communicate through body language. | **0.77** |  |  |  |
| Q18 Uncharacteristic behaviours in a person experiencing dementia are generally a response to unmet needs. | **0.59** |  |  |  |
| Q19 Medications are the most effective way of treating behavioural symptoms of dementia. | **0.33** |  |  |  |
| Q20 People experiencing dementia do not generally have problems making decisions. |  |  | **-0.50** |  |
| Q21 Movement is generally affected in the later stages of dementia. |  |  | **-0.62** |  |
| Q22 People with advanced dementia may have difficulty speaking. |  |  | **-0.29** | 0.24 |
| Q23 People experiencing dementia often have difficulty learning new skills. |  | 0.22 |  | 0.25 |
| Q24 Difficulty eating and drinking generally occurs in the later stages of dementia. |  |  | **-0.48** |  |
| Q25 Daily care for a person with advanced dementia is effective when it focuses on providing comfort. |  |  | **-0.49** |  |

*The factor loadings smaller than 0.20 are not shown; The factor loadings greater than 0.30 are shown in bold.

**Table 2**: Internal consistency of the DKAS subscales

| **Subscale** | **Dutch DKAS scale;**  **N = 655** | | | |
| --- | --- | --- | --- | --- |
|  | **No. of items** | **Range of scores** | **Mean ± SD** | **α** |
| Causes and Characteristics | 7 | 0-14 | 7.77 (2.76) | 0.49^1^ |
| Communication and behaviour | 6 | 0-12 | 5.01 (2.55) | 0.53 |
| Risk Factors and Health Promotion | 6 | 0-12 | 7.85 (3.12) | 0.62^2^ |
| Care considerations | 6 | 0-12 | 3.43 (2.22) | 0.75^3^ |

^1^ Cronbach’s alpha elevated to 0.51 if item 4 was deleted.

^2^  Cronbach’s alpha elevated to 0.65 if item 23 was deleted.

^3^ Cronbach’s alpha elevated to 0.79 if item 14 and 19 were deleted.

*Abbreviations:* α = Cronbach’s alpha, DKAS = Dementia Knowledge Assessment Scale

**Appendix 2.** Measurements of lifestyle related risk factors of dementia

***Physical activity***

Physical activity is calculated based on self-reported questionnaire asking “Thinking about the last two weeks, how many minutes on average per day were you moderate to vigorous physically active?” and “Thinking about the last two weeks, how many days a week did you do muscle and bone strengthening activities?”. The average sum of minutes of moderate to vigorous physical activity per week is reported and frequency of self-reported weekly strength exercise. Physical inactivity is defined as less than 150 minutes moderate to vigorous physical activity per week and less than two times per week doing strength exercises (26).

***Diet***

The MIND diet is a hybrid from the Mediterranean diet and Dietary Approaches to Stop Hypertension (DASH) diet, which has shown to slow down cognitive decline and to decrease the risk of developing dementia (27,28). Adherence to the MIND diet is determined using a food frequency questionnaire (FFQ). For every component two questions were asked “How often did you eat [*component*] in the past month?” and “How many [*serving spoons/table spoon/pieces/portions*] did you take per day?”. The following food components were included in the questionnaire, namely legumes, vegetables, fruit, fish, meat, poultry, nuts, cheese and olive oil. Based on the intake, the adherence to the MIND diet was determined. A score of nine points stands for a good adherence to the MIND diet, zero points for poor adherence (see **Table 1** below).

**Table 1.** MIND-diet scoring table

| **MIND components** | **Recommended quantity** | **Max score** |
| --- | --- | --- |
| Legumes | ≥ 3 serving spoons / week | 1 |
| Other vegetables | ≥ 6 serving spoons / week | 1 |
| Fruit | ≥ 14 servings / week | 1 |
| Fish | ≥ 1 portion / week | 1 |
| Meat | < 4 portions / week | 1 |
| Poultry | ≥ 2 portions / week | 1 |
| Nuts | ≥ 5 servings / week | 1 |
| Cheese | < 1 serving / week | 1 |
| Olive oil | Used as primary oil | 1 |
| **Total score** |  | **9** |

***Alcohol consumption***

Alcohol consumption was measured using the following two questions “How often did you drink alcohol in the past month?” (e.g., no consumption last month, 1 day per month, 2-3 days per month, 1 day per week, 2- 3 days per week, 4-5 days per week, 6-7 days per week) and “How many glasses did you drink on average per day?” (range from zero to more than twelve). Subsequently, the number of glasses of alcohol per week was calculated in order to classify participants into: 1) non-alcohol consumers, 2) low/moderate alcohol consumers or 3) excessive alcohol consumers. Participants adhere to the national recommendations for no to low/moderate alcohol consumption, if participants drink on average one glass or less alcohol per day, without binge drinking (i.e., more than three glasses alcohol per occasion for females and more than four glasses alcohol per occasion for males)) (25).

***Smoking***

Smoking behaviour was assessed with the following two questions: “Did you smoke in the past month?” and “Have you ever smoked a full year?”. Non-smokers are defined as people who did not smoke in past month and never smoked for more than a year. Current smokers are defined as people who reported smoking in the past month. Ex-smokers are defined as people who reported smoking for more than one year in the past, but did not smoke in the past month.

**Appendix 3.** Flowchart of potential participants selected and stratified by age and
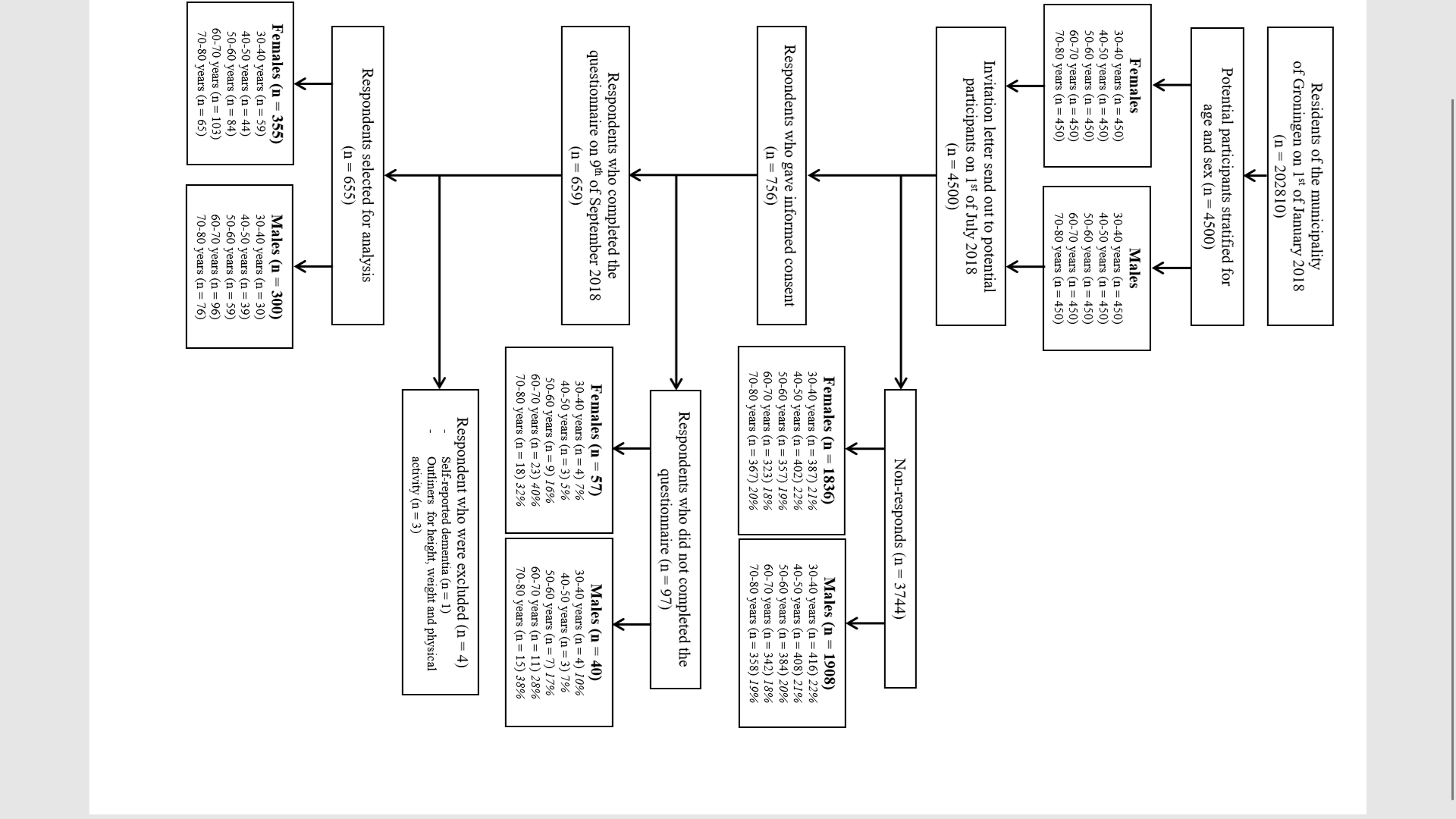
sex

**Appendix 4:** Characteristics of participants stratified by health behaviour status

|  | Physically active | Physically inactive | No to moderate alcohol consumption | Excessive alcohol consumption | Non smoking | Smoking |
| --- | --- | --- | --- | --- | --- | --- |
| N | 264 (40%) | 391 (60%) | 433 (56%) | 222 (44%) | 573 (87%) | 82 (13%) |
| Age in years, mean (SD)^†^ | 56.6 (14.1) | 58.2 (12.8) | 56.8 (13.7) | 59.1 (12.5) | 57.9 (13.5) | 55.4 (12.4) |
| Age groups^§^ |  |  |  |  |  |  |
| 30-45y | 60 (23%) | 69 (18%) | 94 (22%) | 35 (16%) | 111 (19%) | 18 (22%) |
| 45-65y | 105 (40%) | 177 (45%) | 184 (43%) | 98 (44%) | 239 (42%) | 43 (52%) |
| 65-80y | 99 (38%) | 145 (37%) | 155 (36%) | 89 (40%) | 223 (39%) | 21 (26%) |
| Sex, female^§^ | 145 (55%) | 210 (54%) | 269 (62%) | 86 (39%) | 310 (54%) | 45 (55%) |
| Education^§^ |  |  |  |  |  |  |
| Low to middle | 89 (34%) | 170 (44%) | 186 (43%) | 73 (33%) | 218 (38%) | 41 (50%) |
| High | 172 (65%) | 218 (56%) | 242 (56%) | 148 (67%) | 350 (61%) | 40 (49%) |
| Employment, yes^§^ | 141 (53%) | 230 (59%) | 249 (58%) | 122 (55%) | 317 (55%) | 54 (66%) |

**Table 1.** Characteristics of participants stratified by health behaviour status*

Abbreviations: N, number; SD, standard deviation
*Noted in N (%) unless indicated otherwise

**Appendix 5.** Summary of the results of the uni- and multivariable regression analyses

|  | Model 0  OR (95%-CI) | Model 1  OR (95%-CI) | Model 2  OR (95%-CI) |
| --- | --- | --- | --- |
| **Perceived susceptibility (3 items)** | 1.04 (0.95, 1.14) | 0.99 (0.89, 1.10) | 0.96 (0.97, 1.07) |
| **Perceived severity (5 items)** | **1.10 (1.01, 1.20)** | 0.99 (0.93, 1.05) | 1.00 (0.94, 1.07) |
| **Perceived benefits (2 items)** | **1.44 (1.25, 1.66)** | **1.36 (1.15, 1.62)** | **1.33 (1.11, 1.58)** |
| **Perceived barriers (4 items)** | 1.06 (0.98, 1.15) | 1.05 (0.96, 1.15) | 1.03 (0.93, 1.13) |
| **Cues to action (4 items)** | **1.20 (1.11, 1.29)** | **1.11 (1.01, 1.21)** | **1.13 (1.03, 1.24)** |
| **General health motivation (3 items)** | 1.11 (0.99, 1.24) | 1.10 (0.97, 1.25) | 1.14 (0.99, 1.30) |
| **Self-efficacy (2 items)** | **1.25 (1.10, 1.42)** | 0.98, 0.83, 1.16) | 0.93 (0.78, 1.10) |

**Table 1.** The association between MCLHB-DRR subscales and the intention to change physical activity (n=391)

Abbreviations: *OR* odds ratio; *CI* confidence interval
Model 0: Univariable logistic regression analysis

Model 1: Multivariable logistic regression analysis corrected for other MCLHB-DRR subscales
Model 2: Multivariable logistic regression analysis corrected for age, sex, education, employment and other MCLHB-DRR subscales

**Table 2.** The association between MCLHB-DRR subscales and the intention to change diet (n=655)

|  | Model 0  OR (95%-CI) | Model 1  OR (95%-CI) | Model 2  OR (95%-CI) | Model 3*  OR (95%-CI) | |
| --- | --- | --- | --- | --- | --- |
|  |  |  |  | Low to middle  Education  (n=262) | High  Education  (n=393) |
| **Perceived susceptibility (3 items)** | 1.08 (0.99, 1.17) | 1.03 (0.93,1.13) | 1.00 (0.91, 1.11) | 0.91 (0.78, 1.07) | 1.06 (0.93, 1.21) |
| **Perceived severity (5 items)** | 1.05 (0.99, 1.10) | 1.02 (0.96,1.08) | 1.04 (0.97, 1.10) | **1.13 (1.03, 1.24)** | 0.98 (0.89, 1.07) |
| **Perceived benefits (2 items)** | **1.21 (1.07, 1.36)** | 1.11 (0.95,1.29) | 1.07 (0.91, 1.26) | **0.78 (0.60, 0.99)** | **1.41 (1.12, 1.78)** |
| **Perceived barriers (4 items)** | **1.17 (1.08, 1.26)** | **1.13 (1.04, 1.23)** | **1.10 (1.01, 1.21)** | 1.05 (0.91, 1.21) | **1.14 (1.01, 1.28)** |
| **Cues to action (4 items)** | **1.13 (1.06, 1.21)** | **1.05 (0.97, 1.15)** | 1.06 (0.98, 1.16) | 1.14 (0.99, 1.31) | 1.03 (0.92, 1.15) |
| **General health motivation (3 items)** | 0.97 (0.87, 1.08) | 0.97 (0.86, 1.09) | 0.97 (0.86, 1.10) | 0.99 (0.83, 1.18) | 0.97 (0.81, 1.15) |
| **Self-efficacy (2 items)** | **1.17 (1.04, 1.32)** | 1.06 (0.91, 1.25) | 1.05 (0.89, 1.24) | 1.10 (0.85, 1.42) | 1.03 (0.82, 1.30) |

Abbreviations: *OR* odds ratio; *CI* confidence interval
Model 0: Univariable logistic regression analysis

Model 1: Multivariable logistic regression analysis corrected for other MCLHB-DRR subscales
Model 2: Multivariable logistic regression analysis corrected for age, sex, education, employment and other MCLHB-DRR subscales

Model 3: Multivariable logistic regression analysis corrected for age, sex, employment and other MCLHB-DRR subscales (stratified for educational level)

* The interaction term perceived benefits*educational level was statistically significant (p=0.002)

**Table 3.** The association between MCLHB-DRR subscales and the intention to change alcohol consumption (n=222)

|  | Model 0  OR (95%-CI) | Model 1  OR (95%-CI) | Model 2  OR (95%-CI) | Model 3*  OR (95%-CI) | | |
| --- | --- | --- | --- | --- | --- | --- |
|  |  |  |  | 30-45 years  (n=37) | 45-65 years  (n=104) | 65-80 years  (n=81) |
| **Perceived susceptibility (3 items)** | 1.08 (0.95, 1.22) | 1.00 (0.87, 1.16) | 0.97 (0.84, 1.14) | 0.97 (0.54, 1.77) | 1.03 (0.80, 1.31) | 1.02 (0.75, 1.40) |
| **Perceived severity (5 items)** | 1.01 (0.93, 1.09) | 0.96 (0.87, 1.06) | 0.97 (0.87, 1.07) | **2.70 (1.04, 6.97)** | **0.81 (0.67, 0.99)** | 0.92 (0.76, 1.11) |
| **Perceived benefits (2 items)** | **1.43 (1.18, 1.73)** | **1.29 (1.01, 1.66)** | **1.30 (1.00, 1.69)** | **9.59 (1.15, 79.81)^1^** | 0.88 (0.58, 1.34) | 1.55 (0.93, 2.59) |
| **Perceived barriers (4 items)** | 1.02 (0.90, 1.15) | 1.00 (0.88, 1.15) | 1.00 (0.86, 1.15) | 0.98 (0.58, 1.67) | 1.14 (0.92, 1.44) | 0.83 (0.61, 1.13) |
| **Cues to action (4 items)** | **1.23 (1.10, 1.37)** | **1.16 (1.02, 1.34)** | **1.17 (1.02, 1.35)** | 0.52 (0.25, 1.10) | **1.43 (1.12, 1.83)** | **1.48 (1.03, 2.11)** |
| **General health motivation (3 items)** | 1.00 (0.85, 1.17) | 1.03 (0.86, 1.24) | 1.05 (0.86, 1.27) | 2.02 (0.89, 4.60) | 1.20 (0.86, 1.67) | 0.90 (0.65, 1.25) |
| **Self-efficacy (2 items)** | **1.23 (1.02, 1.48)** | 0.96 (0.76, 1.21) | 0.88 (0.68, 1.14) | 0.30 (0.06, 1.38) | 1.08 (0.71, 1.63) | 0.73 (0.45, 1.19) |

Abbreviations: *OR* odds ratio; *CI* confidence interval
Model 0: Univariable logistic regression analysis

Model 1: Multivariable logistic regression analysis corrected for other MCLHB-DRR subscales
Model 2: Multivariable logistic regression analysis corrected for age, sex, education, employment and other MCLHB-DRR subscales

Model 3: Multivariable logistic regression analysis corrected for sex, education, employment and other MCLHB-DRR subscales (stratified for age)

* The interaction term perceived severity*age was statistically significant (p=0.028)

^1^ The relatively small sample of younger participants which were more often highly educated (76%) than the middle-aged (71%) and older participants (58%) (not significant), could have led to the relatively high OR with a broad confidence interval.

**Table 4.** The association between MCLHB-DRR subscales and the intention to change smoking behaviour (n=82)

|  | Model 0  OR (95%-CI) | Model 1  OR (95%-CI) | Model 2  OR (95%-CI) |
| --- | --- | --- | --- |
| **Perceived susceptibility (3 items)** | **1.25 (1.02, 1.53)** | 1.16 (0.91, 1.48) | 1.08 (0.84, 1.39) |
| **Perceived severity (5 items)** | 1.06 (0.94, 1.20) | 1.00 (0.87, 1.15) | 1.05 (0.89, 1.24) |
| **Perceived benefits (2 items)** | 1.14 (0.90, 1.45) | 1.03 (0.71, 1.50) | 0.88 (0.58, 1.34) |
| **Perceived barriers (4 items)** | 0.91 (0.77, 1.07) | 0.82 (0.67, 1.00) | **0.78 (0.63, 0.98)** |
| **Cues to action (4 items)** | 1.16 (1.00, 1.34) | 1.23 (0.97, 1.55) | 1.19 (0.93, 1.52) |
| **General health motivation (3 items)** | 1.07 (0.87, 1.31) | 1.01 (0.80, 1.26) | 1.01 (0.78, 1.33) |
| **Self-efficacy (2 items)** | 1.04 (0.78, 1.38) | 0.86 (0.55, 1.34) | 0.89 (0.54, 1.46) |

Abbreviations: *OR* odds ratio; *CI* confidence interval
Model 0: Univariable logistic regression analysis

Model 1: Multivariable logistic regression analysis corrected for other MCLHB-DRR subscales
Model 2: Multivariable logistic regression analysis corrected for age, sex, education, employment and other MCLHB-DRR subscales

**Appendix 6:** Comparison in number of items between three versions of the MCLHB-DRR scale

| **Subscale** | **Dutch MCLHB-DRR**  **N = 655** | | **English MCLHB-DRR scale**  **N = 617** | | **Turkish MCLHB-DRR**  **N = 220** | |
| --- | --- | --- | --- | --- | --- | --- |
|  | **No. of items** | **Scoring range** | **No. of items** | **Scoring range** | **No. of items** | **Scoring range** |
| Perceived susceptibility | 3 | 3-15 | 4 | 4-20 | 4 | 4-20 |
| Perceived severity | 5 | 5-25 | 5 | 5-25 | 5 | 5-25 |
| Perceived benefits | 2 | 2-10 | 4 | 4-20 | 4 | 4-20 |
| Perceived barriers | 4 | 4-20 | 4 | 4-20 | 4 | 4-20 |
| Cues to action | 4 | 4-20 | 4 | 4-20 | 4 | 4-20 |
| General health motivation | 3 | 3-15 | 4 | 4-20 | 4 | 4-20 |
| Self-efficacy | 2 | 2-10 | 2 | 2-10 | 2 | 2-10 |

MCLHB-DRR = Motivation to Change Lifestyle and Health Behaviours for Dementia Risk Reduction.
